# Protein Tyrosine Phosphatase 1B (PTP1B): A Comprehensive Review of Its Role in Pathogenesis of Human Diseases

**DOI:** 10.3390/ijms25137033

**Published:** 2024-06-27

**Authors:** Dominika Kołodziej-Sobczak, Łukasz Sobczak, Krzysztof Z. Łączkowski

**Affiliations:** 1 Department of Chemical Technology and Pharmaceuticals, Faculty of Pharmacy, Collegium Medicum, Nicolaus Copernicus University, Jurasza 2, 85-089 Bydgoszcz, Poland; d.kolodziej@doktorant.umk.pl; 2 Hospital Pharmacy, Multidisciplinary Municipal Hospital in Bydgoszcz, Szpitalna 19, 85-826 Bydgoszcz, Poland

**Keywords:** PTP1B, diabetes mellitus, obesity, major depressive disorder, Alzheimer’s disease, fatty liver disease, cancer

## Abstract

Overexpression of protein tyrosine phosphatase 1B (PTP1B) disrupts signaling pathways and results in numerous human diseases. In particular, its involvement has been well documented in the pathogenesis of metabolic disorders (diabetes mellitus type I and type II, fatty liver disease, and obesity); neurodegenerative diseases (Alzheimer’s disease, Parkinson’s disease); major depressive disorder; calcific aortic valve disease; as well as several cancer types. Given this multitude of therapeutic applications, shortly after identification of PTP1B and its role, the pursuit to introduce safe and selective enzyme inhibitors began. Regrettably, efforts undertaken so far have proved unsuccessful, since all proposed PTP1B inhibitors failed, or are yet to complete, clinical trials. Intending to aid introduction of the new generation of PTP1B inhibitors, this work collects and organizes the current state of the art. In particular, this review intends to elucidate intricate relations between numerous diseases associated with the overexpression of PTP1B, as we believe that it is of the utmost significance to establish and follow a brand-new holistic approach in the treatment of interconnected conditions. With this in mind, this comprehensive review aims to validate the PTP1B enzyme as a promising molecular target, and to reinforce future research in this direction.

## 1. Introduction

Protein tyrosine phosphatase 1B (PTP1B) is one of the class I non-receptor tyrosine phosphatases, encoded by the *PTPN1* gene. Research on this enzyme began in 1988, after Nicholas K. Tonks et al. isolated this previously unknown phosphatase from human placenta [1,2]. By 1994, an extensive investigation resulted in attainment of the crystallographic structure of the enzyme [3]. This milestone enabled further analysis of its role in regulation of the basic physiology of cells, involving processes such as adhesion, division, growth, mobility, and apoptosis. Before long, it also became apparent that the overexpression of PTP1B induces oncogenesis in ovaries [4]. Moreover, it was determined that the overexpression of PTP1B promotes metastases in the aggressive HER2+ type of breast cancer. This characteristic results from the induction of ErbB2 [5], which is also known as human epidermal growth factor receptor 2 (HER2). ErbB2 plays a significant role in breast carcinogenesis by enhancing the activity of protein tyrosine kinases (PTKs) [6]. Such findings instantly made PTP1B an attractive molecular target for drugging with inhibitors.

However, materialization of this idea proved problematic as there is close structural similarity between various phosphatases present in the human body. Usually, each enzyme is accompanied by at least one close structural homologue. This is exactly the case with PTP1B, which presents significant structural similarity to T-cell protein tyrosine phosphatase (TCPTP), encoded by the *PTPN2* gene. Varying sources state that both enzymes share as much as 72–74% resemblance in the amino acid sequences of their catalytic domains [7,8]. TCPTP is known to regulate inflammatory response, immune response, and tumor development [9]. It is also considered a viable target for cancer immunotherapy with its inhibitors [10,11]. Moreover, despite some differences in mechanisms of action [12], inhibition of both TCPTP and PTP1B affects the regulation of signaling pathways of leptin and insulin [13]. The following is beneficial in pharmacotherapy of diseases such as diabetes mellitus type I (T1DM) [14], diabetes mellitus type II (T2DM), and obesity [15]. Suppressing activity of both phosphatases results in sensitization of insulin receptors, which is desired in therapy of T2DM, but also alleviates the symptoms of T1DM [16]. Additionally, it increases leptin signaling to stabilize glucose metabolism, which prevents hypoglycemic episodes and uncontrolled weight gain. However, despite great similarity between these enzymes, the selectivity of drugs modulating the activity of phosphatases could prove essential for their safety profiles. This became evident as research involving genetically modified mice delivered some alarming evidence. Specifically, the animals without TCPTP perished within 5 weeks from birth [17], while the animals without PTP1B did not [14]. Such findings clearly emphasize the great importance of rational drug design, employed to obtain compounds that are highly selective only towards the specific molecular target. Thankfully, several concepts addressing this issue were already proposed and are discussed in Section 4 of this review.

**Figure 1:**
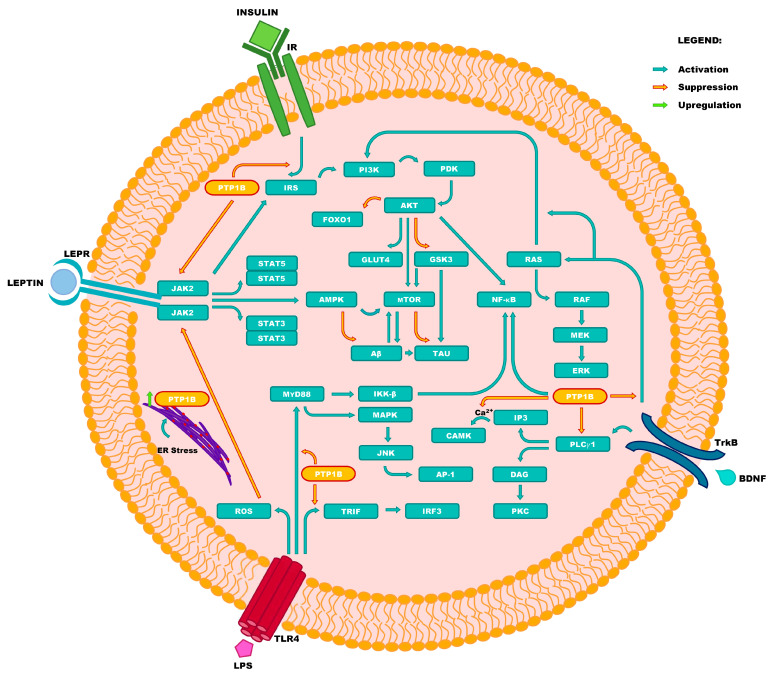
The involvement of PTP1B in pathogenesis of human diseases. Caption: Activation of signaling pathway related to Janus kinase 2 (JAK2), which is initiated by the binding of leptin to its receptor (LEPR), results in regulation of energy expenditure homeostasis, as well as hunger adjustment. The latter results from the induction of signal transducer and activator of transcription 3 (STAT3), which mediates the target gene transcription. Moreover, this pathway is critical in preventing the onset of Alzheimer’s disease (AD) through the inhibition of amyloid β (Aβ) accumulation (prevention of amyloid plaques formation), and inhibition of tau protein (TAU) phosphorylation. Both factors were previously recognized as direct risk factors of AD, and as markers of cognitive function deterioration. Furthermore, the activation of JAK2 is responsible for the induction of the signaling pathway initiated by insulin receptor (IR) activation, which is also known as the insulin receptor substrate/phosphoinositide 3-kinase/protein kinase B (IRS/PI3K/AKT) pathway. The activation of the IRS/PI3K/AKT pathway leads to the suppression of the forkhead box protein O1 (FOXO1), which results in the inhibition of gluconeogenesis. Moreover, the activation of the IRS/PI3K/AKT pathway inhibits glycogenesis by the suppression of glycogen synthase kinase 3 (GSK3), and determines cognitive function by regulating the survival and neuroplasticity of neurons. This pathway is also related to the cascade initiated by the activation of tropomyosin receptor kinase B (TrkB), by the direct or indirect activation of the rat sarcoma virus (G protein)/rapidly accelerated fibrosarcoma kinase(s) (RAS/RAF) pathway. The RAS/RAF pathway is responsible for cell growth, survival, and neuroplasticity; along with phospholipase C gamma 1 (PLC*γ*1) activation resulting in the expression of Ca^2+^/calmodulin-dependent protein kinase(s) (CAMK) and protein kinase C (PKC), which are also responsible for neuroplasticity. The activation of toll-like receptor 4 (TLR4) induced by lipopolysaccharides (LPSs), plays a dual role in the pathogenesis of fatty liver disease. On one hand, the accumulation of LPSs promotes the synthesis of proinflammatory cytokines induced by reactive oxygen species (ROS). On the other, by inhibiting the JAK2/STAT3 pathway, PTP1B prevents the release of proinflammatory cytokines from macrophages related to the TIR-domain-containing adapter-inducing interferon-beta (TRIF) and myeloid differentiation primary response 88 protein (MyD88).

Finally, benefiting from the latest findings, this review provides an updated and comprehensive summary of the involvement of PTP1B expression-related disorders in the pathogenesis of human diseases. Moreover, the scope of this review deliberately involves all—even supposedly unrelated—branches of medicine. Motivated by a strong belief that it is important to establish a brand-new holistic approach towards the therapy of human diseases, we strive to present the already identified therapeutic targets in a broader context; with the goal of opposing the dated and isolated approach, which often results in dangerous polypharmacy [18]. Figure 1 presents the overview of complex associations existing between signaling pathways involving the PTP1B enzyme, with all the direct and indirect targets additionally listed in Table 1. Last, but not least, we intend to demonstrate that overcoming the present difficulties with attainment of safe and effective PTP1B inhibitors may promptly result in critical advancements in future pharmacotherapy of several important and abundant diseases. Therefore, the main goal of this work is to encourage and aid intensified effort towards imminent achievement of this goal.

## 2. Role of PTP1B in Pathogenesis of Human Diseases and Prospects of Therapy with Inhibitors of the Enzyme

### 2.1. Diabetes Mellitus and Obesity

In 2021, approximately 537 million adults and over 1 million children worldwide were affected by diabetes [19]. This accounts for roughly 6.9% of the world population. Recent guidelines for prevention and therapy of diabetes emphasize the fundamental role of a healthy lifestyle, employed by the means of a balanced diet and regular physical exercise [20]. If the healthy lifestyle is unable to maintain appropriate control of blood glucose, then the subsequent pharmacotherapy involves administration of insulin in T1DM, and hypoglycemic drugs in T2DM. However, there are numerous complications associated with the present-day antidiabetic treatment. Inadequate administration of insulin creates a risk of life-threatening hypoglycemia [21], may lead to eventual insulin resistance due to desensitization of insulin receptors [22], and increases the risk of cardiological complications such as coronary artery disease or lipotoxic cardiomyopathy [23]. Similarly, currently available oral hypoglycemic drugs are well known to cause numerous side effects [24]. Metformin, which is recommended as the first-choice medication in pharmacotherapy of T2DM [20], is also among the most popular drugs in the world and is included in the World Health Organization (WHO) model list of essential medicines (EMLs) [25]. Furthermore, according to the latest data it was the second most prescribed medicine in the United States—with over 91 million prescriptions in 2021 alone [26]. Yet, as many as 5% of all patients are unable to tolerate this drug, and suffer from gastrointestinal disorders such as abdominal pain, diarrhea, and flatulence [27]. Moreover, metformin therapy is frequently (in up to 30% of the cases) associated with vitamin B_12_ deficiency, which may eventually result in anemia [27]. According to the guidelines, alternative first-line recommendations are dipeptidyl peptidase-4 (DPP-4) inhibitors (“gliptins”), pioglitazone, sodium-glucose linked transporter 2 (SGLT2) inhibitors (“gliflozins”), or sulfonylureas [20]. Among these drugs, some of the most popular choices include (with the following information presented according to the latest data available for US market): sitagliptin (>8.5 million prescriptions [26]); empagliflozin (>8.4 million prescriptions [26]); pioglitazone (>4.5 million prescriptions [26]); and dapagliflozin (>2.6 million prescriptions [26], > USD 5.9 billion sales [28]). However, there are certain drawbacks of each option. Sitagliptin may cause severe and disabling joint pain [29], empagliflozin and dapagliflozin are typically associated with an increased risk of urinary tract infections [30] and are proven to increase the risk of potentially life-threatening diabetic ketoacidosis [30,31], while pioglitazone may increase the risk of bladder cancer [32].

Obesity treatment is another thriving trend in drug discovery, with the market expected to grow over 6-fold within the next 10 years [33]. Current anti-obesity blockbusters include several glucagon-like peptide-1 (GLP-1) receptor agonists, drugs initially developed as antidiabetic medications, such as semaglutide (>8.1 million prescriptions [26], >USD 13.8 billion sales [28]) and liraglutide (>3.2 million prescriptions [26]). Unfortunately, many patients (up to 77% [34,35,36]) suffer from gastrointestinal disruptions such as constipation, diarrhea, and vomiting. Some central nervous system stimulants are also popular choices for obesity treatment, with phentermine being the primary example (>2.1 million prescriptions [26]). This methamphetamine isomer is used in monotherapy, or in combination with topiramate, a carbonic anhydrase inhibitor. Yet some nervous system-related adverse effects including dysgeusia, paresthesia, dry mouth, attention disturbance, and irritability have been observed following such treatment [37].

On the contrary, the newly developed PTP1B inhibitors offer unique therapeutic prospects, all thanks to the sensitization of insulin receptors and direct increase in leptin activity in the hypothalamus.

The role of PTP1B in the pathogenesis of diabetes mellitus results predominantly from dephosphorylation of the insulin and leptin receptors [38]. This process decreases their sensitivity and has two major implications. Firstly, desensitization of the IR is recognized as a crucial factor in the development and progression of T2DM [39]. As such, PTP1B is one of the key negative regulators of the IR signaling pathway [40]. Moreover, the overexpression of PTP1B decreases the level of GLUT4 on the surface of the cells [41]. Whereas under normal conditions insulin binding to the IR induces the conformational changes that transport GLUT4 toward the surface of the cells [38], in this case, as the physiological function of GLUT4 is to facilitate the diffusion of the circulating glucose into the cells, the effect induced by PTP1B limits the rate of this process and prevents it from lowering the blood glucose level. Therefore, prolonged overexpression of PTP1B could result in a state of hyperglycemia, which plays a fundamental role in the onset of T2DM. Secondly, PTP1B also desensitizes the leptin receptor and limits one of the most important regulatory roles of leptin, which is to decrease the blood glucose level [42,43]. Finally, the experimental deletion of PTP1B was shown to decrease the incidence of significant diabetic complications such as heart failure, cardiovascular pathologies, retinopathy, nephropathy, and diabetic foot ulcers [40].

Apart from its auxiliary input in the prevention of T2DM, leptin expresses its action by counteracting increases in body weight, even despite a fat-rich diet, by a mechanism of increased glucose reuptake into the brown adipose tissue and by reduced glucose production in the liver [43]. Therefore, leptin is endowed with a critical role in controlling body weight and preventing obesity. Moreover, leptin resistance was also described as a consequence of PTP1B overexpression [44], with the proposed explanation being the inhibition of leptin signaling pathways by PTP1B. More specifically, by dephosphorylation of Janus kinase 2 (JAK2) and signal transducer and activator of transcription 3 (STAT3). The JAK2 enzyme is associated with the active site of the leptin receptor, and the decreased activity of this kinase eventually results in desensitization of the receptor [45]. Therefore, pharmacotherapy with PTP1B inhibitors is intended to restore adequate leptin activity and allow control over blood glucose levels.

Considering the common links between the pathogeneses of diabetes mellitus and obesity, a healthy lifestyle supported by pharmacotherapy with PTP1B inhibitors should offer effective treatment of both diseases. This novel class of drugs enables control of blood glucose without the risk of hypoglycemia (in T1DM), while simultaneously facilitating the effort of weight reduction (as distinctive target, or as an aid in T2DM therapy). Validating this theory, there are numerous examples of PTP1B inhibitors being used in therapy of T2DM and obesity.

**Figure 2:**
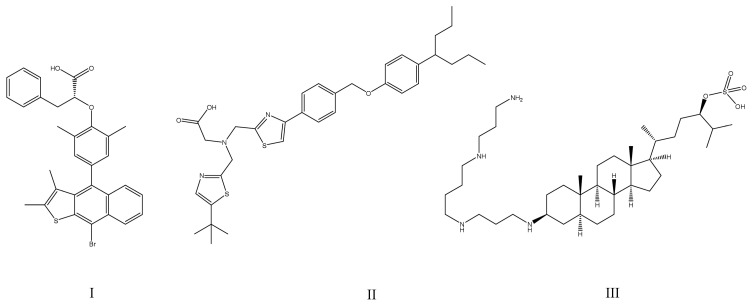
Chemical structures of small-molecule PTP1B inhibitors that entered clinical trials: (**I**)—ertiprotafib; (**II**)—JTT-551; (**III**)—trodusquemine (MSI-1436).

Prior efforts in this field, especially the PTP1B inhibitors that entered clinical trials (ertiprotafib (IC_50_ = 1.6–29 μM [46]), trodusquemine (MSI-1436; IC_50_ = 1 μM [47]), JTT-551 (IC_50_ = 0.22 μM [48]), TTP-814, as well as IONIS (ISIS) 113715 (IC_50_ < 10 nM [49]) and IONIS (ISIS) PTP1BRx), were discussed elsewhere [38,50,51]. Unfortunately, the trials involving all compounds except for the IONIS PTP1BRx were discontinued [50]. The chemical structures of the mentioned small molecules are presented in Figure 2, while the sequences of the synthetic oligonucleotides are presented in Table 2. Very recently, the main strategies employed to inhibit PTP1B (orthosteric, allosteric, and bidentate inhibition, as well as *PTPN1* gene silencing) were described by Coronell-Tovar et al. [52]. Whereas the existing challenges associated with targeting PTP1B, state-of-the-art PTP1B inhibitors, as well as some future directions for regulation of enzyme activity, were covered by Delibegović et al. [53].

**Figure 3:**
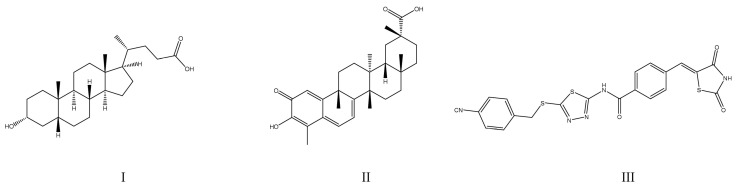
Chemical structures of PTP1B inhibitors investigated for the therapy of diabetes mellitus and obesity: (**I**)—lithocholic acid (reference drug); (**II**)—celastrol; (**III**)—MY17.

Additionally, within recent months some very potent PTP1B inhibitors have been proposed as drug candidates for T2DM treatment. These drugs include 41 derivatives of thiazolidine-2,4-dione (with IC_50_ values as low as 0.41 ± 0.05 μM for MY17) [54], in comparison with the reference lithocholic acid (IC_50_ = 9.62 ± 0.14 μM). The drugs were assessed with HepG2 (human hepatoma) cells and in vivo by oral administration to mice suffering from diabetes mellitus. The most active compound (MY17) was a reversible, noncompetitive inhibitor of PTP1B. Given orally, these drugs are expected to reduce insulin resistance, reduce blood glucose levels, as well as to improve glucose tolerance and dyslipidemia [54]. Other example are the lipidated BimBH3 peptide analogues with PTP1B/TCPTP dual inhibitory activity, which could be administered once a week in low doses (IC_50_ = 0.5 μmol/kg). The proposed compounds were tested in mouse models of T2DM and were additionally investigated to confirm cell permeability [55]. Also of particular interest is a celastrol (IC_50_ = 2.1 μM), a natural pentacyclic triterpene derived from traditional Chinese medicine, which was shown to promote weight loss as a dual PTP1B/TCPTP inhibitor. The following was demonstrated in vivo in a diet-induced obese (DIO) mouse model [56]. The chemical structures of the compounds discussed in this section are presented in Figure 3.

### 2.2. Alzheimer’s Disease

Alzheimer’s disease (AD) is a neurodegenerative disease and one of the most common types of dementia. AD mostly affects the elderly population (that is, above 65 years old), as over 95% of all cases involve the so-called late-onset Alzheimer’s disease (LOAD). Although the exact causes of LOAD remain unestablished, some of the leading theories associate the disease with detrimental environmental factors [57], as well as with the genetic burden resulting from the presence of the apolipoprotein E4 (APOE4) allele [58]. In cases of early-onset Alzheimer’s disease (EOAD), the first symptoms usually occur much before the age of 65 [59]. The common course of both types of AD includes the gradual decline in cognitive functions including short-term memory and executive functions involving speech and communication skills [60].

The cellular pathomechanism of AD involves accumulation of amyloid β (Aβ) caused by autosomal dominant mutations in amyloid precursor protein genes [61], in particular presenilin 1 (PSEN1) and presenilin 2 (PSEN2) [62]. Moreover, the presence of the APOE4 allele was determined as a genetic factor increasing the risk of AD’s onset [63]. Expression of this allele results in the amassment of unsaturated fatty acids and in diminished Aβ clearance. These metabolic alterations lead to neuronal degradation [64]. Additionally, intracellular accumulation of selected other lipids creates a risk of aggravating the condition [65,66,67].

Yet, it needs to be emphasized that genetically determined AD comprises only a small percentage of all cases [68,69]. Therefore, environmental factors determined by lifestyle are the main cause of the disease. Among these factors, the most important are exposure to toxic metals and an unbalanced diet. Particular danger is posed by lead, as it easily penetrates the brain–blood barrier and induces the degradation of myelin sheaths. This causes a decline in cognitive and motor functions, as well as hindering learning and memory processes [70]. Lead exposure additionally creates a risk of triggering AD by promoting Aβ aggregation, which was observed in some in vivo studies [71]. Moreover, the important study of Schwartz et al. provided evidence for the decline in cognitive functions in individuals with prolonged lead exposure and with the simultaneous presence of the APOE4 allele [72]. Also of interest is the neurotoxicity of aluminum and zinc. While aluminum promotes oxidative stress [73], zinc induces intracellular accumulation of Aβ [74] and the generation of amyloid plaques [75]. Another concern is raised by the quality of diet, which affects all aspects of our life, including the potential onset of AD. Excessive caloric supply results in obesity, induces T2DM, and increases insulin resistance, as well as increasing Aβ accumulation [76].

Considering all the above, PTP1B overexpression emerges as a critical factor behind the onset of AD. It also constitutes a common link between the genetic and environmental risk factors of the disease. And while the genetic knockout of PTP1B does not result in a reduction in the density of amyloid plaques, it does reduce their size [77]. Additionally, the neuron-specific ablation of PTP1B inhibits neuronal damage in the hippocampus, protecting the cognitive functions of the brain. It was also determined that obesity is associated with a decrease in the volume of gray matter of the brain, and this structure was previously determined to govern the cognitive functions [78,79,80,81,82]. However, the discussed mechanism is much more intricate. For example, a particular type of fat-rich diet, which is especially rich in docosahexaenoic acid (DHA), is not detrimental for health but has a neuroprotective effect and prevents the development of moderate AD [83].

Currently there is no effective causal therapy of AD; such a therapy would be capable of controlling metabolic alterations to prevent progression of neurodegeneration and preferably to reverse the existing neuronal damage. Therefore, lifestyle changes provide the only option—an approach based on prevention. This is where PTP1B inhibitors emerge as novel therapeutics, which can be used to directly prevent the neuronal damage caused by intracellular accumulation of Aβ, but also indirectly by suppressing the progression of the disease by facilitating control over body weight.

**Figure 4:**
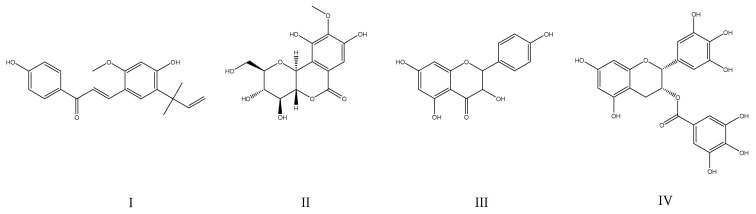
Chemical structures of natural PTP1B inhibitors investigated for therapy of Alzheimer’s disease: (**I**)—licochalcone A; (**II**)—bergenin; (**III**)—kaempferol, (**IV**)—epigallocatechin-3-gallate.

Proving this concept are some active ingredients isolated from traditional medicinal herbs, which were already proven in the therapy of AD and that were also identified as PTP1B inhibitors. Among them was a natural PTP1B inhibitor, licochalcone A (IC_50_ = 19.1 µM ± 0.1). This flavonoid, present in the roots of the plants from the Glycyrrhiza species, is commonly used in traditional Chinese medicine and was determined to enhance cognitive function through the BDNF-TrkB pathway, reduce the formation of amyloid plaques, modulate the brain insulin receptor by inhibition of c-Jun N-terminal kinase 1, and prevent neuroinflammation. In addition to inhibition of PTP1B, licochalcone A also acts as an acetylcholinesterase inhibitor and increases acetylcholine levels in the brain to enhance the cognitive capacity of individuals affected by AD [84]. Other examples include epigallocatechin-3-gallate (IC_50_ = 103.8 ± 10.1 μM [85]), present in green tea, which was found to prevent neurological pathologies such as cognitive loss. For their study, the authors used a well-established preclinical mixed model of AD and T2DM based on APPswe/PS1dE9 (APP/PS1) mice fed with a high-fat diet. The cognitive performance of the APP/PS1 mice was compared to a control group of C57BL/6 wild-type mice. Supplementation with epigallocatechin-3-gallate was also shown to mitigate neuroinflammation through a decrease in astrocyte reactivity and TLR4 expression [86]. Whereas another research group used a rat-based amnesia model induced by scopolamine, as well as a Wistar rat-based model of AD induced by streptozotocin, to demonstrate that bergenin (IC_50_ = 157 µM [87]) has neuroprotective effects [88]. Also, with the use of diabetic rats that had cognitive deficits induced by a high-glucose and high-fat diet, as well as with streptozotocin, a natural PTP1B inhibitor named ferulic acid was shown to attenuate diabetes-induced cognitive impairment [89]. Finally, the flavonoids from mulberry leaves were also investigated, as this plant is well known from its hypoglycemic and antioxidative properties, which can be used for the indirect treatment of AD. The study used network pharmacology, molecular dynamics simulation, and cellular assays (with HepG2 cells) to identify kaempferol (IC_50_ = 279.23 μM) as the most active PTP1B inhibitor originating from this plant [90]. Natural compounds investigated as therapeutics for AD are presented in Figure 4.

### 2.3. Major Depressive Disorder

According to the latest edition of the Diagnostic and Statistical Manual of Mental Disorders (DSM-5), major depressive disorder (MDD) can be defined as a prolonged feeling of anhedonia, helplessness, and sadness. Other criteria involve the duration of time exceeding 2 weeks and presence of five or more out of nine severity markers [91]. Symptoms of MDD manifest ambiguously, but almost always impair the professional and social lives of the affected. Concerning the cause of MDD, several distinct risk factors were identified so far, including genetic predisposition (in up to 37% of all cases) [92]; traumatic events [93]; hormonal changes; and the detrimental impact of environmental stress [94].

Among the multitude of theories regarding the onset of MDD, there are two leading explanations. The first one, known as the neurotransmitter deficiency hypothesis, revolves around the excessive activity of the monoamine oxidase A (MAO-A) enzyme—a condition caused by the overexpression of the *MAOA* gene. Subsequently, it results in overly extensive catabolism and the eventual shortage of catecholamines [95]. As therapy with MAO-A inhibitors is effective in most cases, this seems to validate this hypothesis. Yet, MAO-A inhibitors are not effective in up to 30% of the remaining patients [96]. At the same time, Sun et al. reported that high expression of the *MAOA* gene decreases activity in the hippocampus, and increases cortisol levels, which affects the regulation of the stress reaction [97]. This mechanism provides a common link with the second popular theory. According to this alternative hypothesis, MDD occurs due to the dysfunction of the hypothalamic–pituitary–adrenal (HPA) axis [98]. With correct HPA-axis function, cortisol released as a response to a stressful situation downregulates further hormone secretion by a mechanism of negative feedback. Malfunction of this mechanism is believed to cause MDD. Evidence supporting this hypothesis is provided by observations of patients diagnosed with MDD, where reduced neurogenesis and atrophy of the hippocampus were both present. The authors explain these findings as a result of an increased cortisol level, but also of decreased levels of brain-derived neurotrophic factor (BDNF) [99] and LIM domain only 4 (LMO4) proteins [100].

BDNF and LMO4 are two molecules of particular interest considering their involvement in the pathogenesis of MDD, but also because of their relation to PTP1B in this process. The antidepressant activity of BDNF results from its ability to increase neural flexibility [101]. Edwards et al. demonstrated that a model PTP1B inhibitor (phosphatidic acid) was able to increase phosphorylation of TrkB (BDNF receptor in hippocampus), which restored correct neurogenesis and normalized the behavior of mice with MDD [102]. The second molecule, LMO4, acts as an endogenous PTP1B inhibitor with an anxiolytic action. However, the efficacy of PTP1B inhibition by LMO4 can be reduced by stress [100]. Additionally, LMO4 affects the insulin and leptin signaling pathways by the regulation of PTP1B activity [103,104]. This creates a common link between MDD and previously described metabolic disorders. Moreover, the current estimates indicate that 18% of men and 28% of women diagnosed with diabetes are simultaneously suffering from MDD [105]. Additionally, MDD is considered to be a major risk factor for T2DM, increasing the probability of its onset by 37% [106]. Leptin, which has a fundamental role in the pathogenesis of obesity, also affects a broad spectrum of depressive disorders by the regulation of the HPA axis, and by promoting the activity of BDNF [107].

Considering the above, prevention of metabolic disorders accompanying depression should always be addressed, with parallel attention given to the mental well-being of the patients. In particular, novel PTP1B inhibitors offer a combined prevention and treatment of MDD by the reinforcement of BDNF activity through two distinct mechanisms. Firstly, by the direct prevention of dephosphorylation of BDNF receptors (TrkB), as well as indirectly by increasing the leptin activity to normalize the HPA-axis function. Moreover, drugs acting as PTP1B inhibitors could enhance the endogenous activity of LMO4, a natural PTP1B inhibitor, but could also treat the symptoms of T2DM, a condition often associated with MDD.

Indeed, some PTP1B inhibitors were already investigated in the therapy of MDD. For example, the insufficient expression of microRNA miR-144 was identified as a problem in mice with MDD. Subsequent treatment by injection of Lentivirus with miR-144 overexpressing vector (LV-miR-144) inhibited the expression of PTP1B and provided anti-depressive effects [108].

### 2.4. Fatty Liver Disease

One of the latest trends in drug discovery involves the treatment of fatty liver disease, a condition triggered by excessive PTP1B activity and defined as an accumulation of triglycerides in over 5% of all hepatocytes. Based on its pathogenesis, it is possible to distinguish two separate variants of the disease: alcohol-related fatty liver disease (ALD) and non-alcoholic fatty liver disease (NAFLD). NAFLD is also described as metabolic-associated fatty liver disease (MAFLD), as it is neither related with excessive alcohol consumption, nor with the misuse of other hepatotoxic agents including drugs.

ALD is caused by the disruption of lipopolysaccharide (LPS) management, resulting in inflammation. LPSs can be biosynthesized by intestinal microflora, or can be supplied in food [109] and are primarily responsible for the natural immune response [110,111]. However, if LPSs enter blood circulation through damaged intestinal mucosa, which could result from excessive alcohol consumption, they create a risk of sepsis [112] and trigger prolonged inflammation in ALD [113]. Protracted inflammation caused by alcohol metabolism and endotoxins processed in the liver activates macrophages, which release proinflammatory cytokines, regarded as a direct cause of ALD [114]. Additionally, the activated macrophages were identified to be involved in transportation of reactive oxygen species (ROS), which, along with cytokines, regulate the JAK2/STAT3 signaling pathway [115,116] and the activity of NF-κB [117]. As was previously mentioned in the context of obesity, PTP1B is also involved in the regulation of the JAK2/STAT3 signaling pathway [118]. Furthermore, PTP1B also affects the release of proinflammatory cytokines [118], which was described in detail by Hsu et al. [119]. The authors presented a mechanism, whereby liver damage is prevented by decreased PTP1B expression. This provided evidence for the involvement of PTP1B overexpression in the onset of ALD [119].

The second type of fatty liver disease, which is unrelated to alcohol misuse, has a generalized metabolic syndrome as its main risk factor [120]. Therefore, hepatic insulin resistance, obesity, and T2DM all play essential roles in the onset of NAFLD/MAFLD [121]. All of these factors were previously discussed in their relation to PTP1B overexpression. The disruption of insulin signaling in the liver may result from a fat-rich diet, or from the lipids being ectopically accumulated in tissues. Moreover, disrupted insulin signaling exacerbates liver inflammation [122], which may eventually result in non-alcoholic steatohepatitis (NASH) [123]. This grave condition can further progress into liver cirrhosis, fibrosis, or hepatocellular carcinoma (HCC) [124]—the third most lethal type of cancer in 2022 [125]. Additionally, endoplasmic reticulum (ER) stress induced by obesity [126,127] increases the risk of developing insulin resistance [128]. Inhibition of ER stress can be achieved by the attenuation of PTP1B expression, and could prevent the progression of NAFLD [129]. PTP1B ablation not only prevents the aggravation of the disease, but also enables the regression of NASH and has a fundamental role in the regeneration of the damaged hepatocytes [130]. However, the role of PTP1B in the progression of the disease is equivocal, as it also inhibits the signaling pathway related to toll-like receptor 4 (TLR4) and prevents the release of proinflammatory cytokines from macrophages [131].

Altogether, PTP1B inhibitors offer new and interesting opportunities for prevention and treatment of fatty liver disease. Primarily, because PTP1B inhibitors prevent liver damage by decreasing the release of proinflammatory cytokines from macrophages. But also, by the reduction in ER stress. Simultaneously targeting both of these factors is crucial for the effective treatment of various types of fatty liver disease.

**Figure 5:**
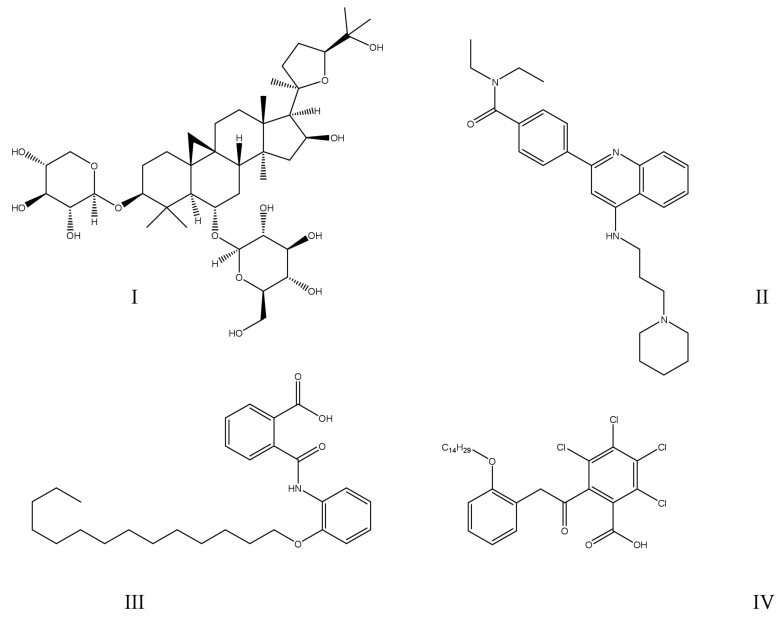
Chemical structures of experimental PTP1B inhibitors investigated for the therapy of fatty liver disease: (**I**)—astragaloside (**IV**); (**II**)—compound **23**; (**III**)—CX08005; (**IV**)—WS090152.

Several PTP1B inhibitors have already been investigated in the treatment of fatty liver disease models, with promising results. These include compound WS090152 (IC_50_ = 0.34 μM), which was found to ameliorate the symptoms of disease by increasing insulin sensitivity and by decreasing hepatic lipogenesis in C57BL/6J mice fed a high-fat diet [132]; CX08005 (IC_50_ = 0.75 ± 0.07 μM), which was shown to attenuate hepatic lipid accumulation and microcirculation dysfunction in both KKAy (a cross between diabetic KK and lethal yellow (Ay) mice) and DIO mice [133]; and astragaloside (**IV)** (IC_50_ = 10.34 ± 0.54 μM), which effectively reduced the triglyceride accumulation in HepG2 cells [134]. Moreover, trodusquemine (IC_50_ = 1 μM [47]), a natural spermine–cholesterol adduct, as well as the newly synthesized quinoline-based compound **23** (N,N-diethyl-4-(4-((3-(piperidin-1-yl)propyl)amino)quinolin-2-yl) benzamide) were both beneficial in alleviating liver lipotoxicity and in the management of NAFLD. The following was likewise established with the use of HepG2 cells [135]. The chemical structures of PTP1B inhibitors that were investigated in the treatment of fatty liver disease are presented in Figure 5.

### 2.5. Cancers

Previously, the inhibition of PTP1B was portrayed as advantageous in the treatment of various medical conditions. However, the consequences of reduced PTP1B activity for the progression of cancers are not unequivocal. Some studies delivered promising reports. For example, concerning the impediment of ovarian cancer progression by inhibition of PTP1B [4], which was mediated by negative regulation of IL-13 receptor α2 (IL13Rα2). Overexpression of this receptor is known to stimulate the progression of ovarian cancer [136]. Other cancer types associated with excessive IL-13 activity include pancreatic cancer [137]; colorectal cancer (CRC) [138]; glioblastoma multiforme (GBM) [139]; renal cell carcinoma [140]; mesothelioma [141]; and malignant melanoma [142]. In addition, the extensive stimulation of IL13Rα2 promotes metastases of breast cancer into the brain [143] and into the lungs [144], but also a metastasis of CRC into the liver [145]. Nevertheless, it should be stressed that the signaling pathway coupled with IL13Rα2 is only one of several existing mechanisms responsible for regulation of the progression of these cancer types. Remarkably, all the listed cancer types are also associated with the overexpression of PTP1B. For example, in the case of breast cancer, PTP1B is known to regulate IL13Rα2. PTP1B is also known to modulate the effects of ErbB2 and PTK [5,6], which is especially important as overexpression of ErbB2 and PTK manifests in as much as 72–90% of all breast cancers [5,146]. Moreover, the amplification of the *PTPN1* gene was determined to be a marker of a severe course of the disease, determining worse prognosis in breast cancer [147], gastric cancer [148], and CRC [149]. The latest studies also reported that overexpression of PTP1B promotes the progression of prostate cancer [150].

On the contrary, oncogenesis resulting from the inhibition of PTP1B (or the knockout of the *PTPN1* gene) was also reported. In particular, the absence of the PTP1B enzyme was established as a critical factor in the process of excessive lymphopoiesis. Additionally, the lack of PTP1B is known to disrupt lymphocyte B function by triggering their accumulation in tissues, which eventually results in the increased incidence of B-cell lymphomas [151]. Furthermore, PTP1B suppresses the progression of esophageal adenocarcinoma by the regulation of the JAK2/STAT3 signaling pathway [152,153]. Therefore, the absence of this enzyme is bound to aggravate the course of the disease.

Considering the multitude of favorable effects resulting from the inhibition of PTP1B, it is not surprising that this enzyme is considered to be a very promising therapeutic target. However, reports providing evidence for both suppression and induction of tumor formation (all depending on the specific tissues and the genetic background [154]) necessitate increased vigilance concerning the use of PTP1B inhibitors in cancer therapy. Full understanding of all consequences resulting from the inhibition of PTP1B, especially the long-term effects of such treatment, is yet to come. In the meantime, intensified research focusing on the signaling pathways regulated by PTP1B, as well as the development of even more selective enzyme inhibitors, should continue to be pursued—as the goal of enhanced cancer therapy remains as important as ever.

**Figure 6:**
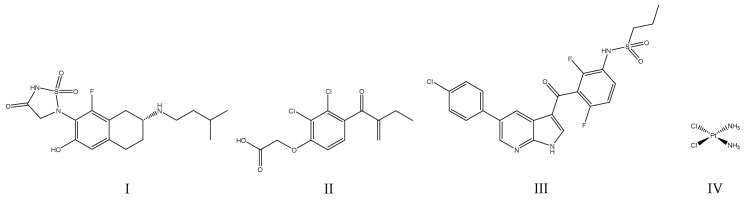
Chemical structures of PTP1B inhibitors and drugs with PTP1B inhibitory activity which have been investigated in cancer therapies: (**I**)—osunprotafib (ABBV-CLS-484); (**II**)—ethacrynic acid; (**III**)—vemurafenib; (**IV**)—cisplatin.

Nevertheless, PTP1B inhibitors are currently popular investigational drugs in anticancer therapies. Trodusquemine (IC_50_ = 1 μM [47]), previously described in this review as effective in T2DM and NAFLD [135], was also employed in the treatment of breast cancer. The properties of this experimental drug were demonstrated by inhibition of tumorigenesis in xenografts and retraction of metastasis in the NDL2 mouse model of breast cancer [155]. Whereas osunprotafib (ABBV-CLS-484; IC_50_ = 2.8 nM), a very active dual PTP1B/TCPTP inhibitor with nanomolar activity is currently undergoing clinical evaluation for cancer immunotherapy in patients with advanced solid tumors [11,156]. Moreover, several well-renowned drugs were lately identified as having auxiliary action as PTP1B inhibitors. The examples include cisplatin, which can impair the enzymatic functions of PTP1B [157]; vemurafenib, which was licensed for the treatment of non-resectable metastasized melanoma in humans [158], and based on a study with colorectal cancer lines HT29 and HCT116 it was also found to down-modulate the phosphatases (including PTP1B) serving as aggressiveness mediators in this type of cancer [159]; as well as ethacrynic acid, which restrained the growth of DU145 prostate carcinoma cells in xenografted mice by modulation of the PTP1B, leading to the inhibition of STAT3 activity [160]. The chemical structures of these drugs are presented in Figure 6.

**Figure 7:**
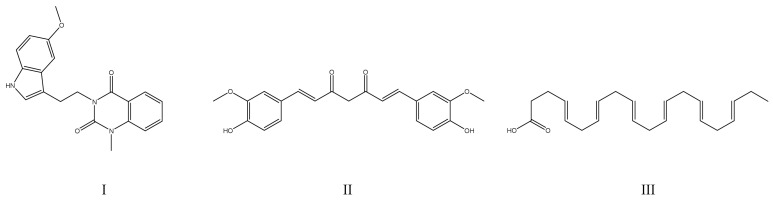
Chemical structures of natural PTP1B inhibitors investigated in cancer therapies: (**I**)—10-methoxygoshuyuamide (**II**); (**II**)—curcumin; (**III**)—docosahexaenoic acid.

In addition, some traditional medicinal plants with previously proven anticancer activity contain the natural PTP1B inhibitors 10-methoxygoshuyuamide (**II**) (IC_50_ = 75.8 μM), an indole alkaloid isolated from the fruits of *Evodia rutaecarpa* which inhibited four human cancer cell lines (MCF-7, Hepg-2, A549, and SHSY-5Y) [161]; curcumin (IC_50_ = 100 μM [162]), a popular natural compound, along with several of its derivatives featuring a 4-piperidone ring was proposed as a breast cancer chemotherapeutic based on an investigation involving breast cancer MCF-7 and MDA-MB-231 cell lines and the human keratinocyte HaCaT cell line [163]; and docosahexaenoic acid (IC_50_ = 173.5 ± 25.5 µM), an essential polyunsaturated fatty acid that can be found in select fishes, was shown to inhibit MCF-7 breast cancer cells [164]. Also, several catechins present in green tea leaves decreased the viability of MCF-7 breast cancer cells and were identified as PTP1B inhibitors, namely: epigallocatechin (IC_50_ = 103.8 ± 10.1 μM), epigallocatechin gallate (IC_50_ > 500 μM), epicatechin (IC_50_ > 500 μM), and epicatechin gallate (IC_50_ > 500 μM) [85]. The chemical structures of natural PTP1B inhibitors with anticancer activity are presented in Figure 7. Lastly, similarly to miR-144, employed for the treatment of MDD [108], a fellow microRNA—miR-34c—was shown to inhibit the proliferation of human glioma by targeting PTP1B in severe combined immune deficiency (SCID) mice with xenografted tumors [165].

### 2.6. Other Diseases

Apart from the previously discussed diseases, ongoing studies concerning PTP1B overexpression offer brand new and often unique applications for the recently introduced PTP1B inhibitors. One such example is their use in schizophrenia-like symptoms manifesting as behavioral, cognitive, and emotional disorders in mice with LMO4 deficiencies. Similarly to the previously described pathomechanism of MDD, this condition results from the inhibition of TrkB caused by the overexpression of PTP1B [166]. This observation is especially important, as currently there is no general consensus regarding the actual pathomechanism of schizophrenia. Perhaps the answer is related to the previously overlooked role of phosphatases. If this could be confirmed, then completely new opportunities for treatment of this disorder would be created.

Moreover, the neuroprotective effects of PTP1B inhibitors, such as the improved regulation of neuronal κB-binding factors (NκBFs) and the attenuation of ER stress were described by Feng et al. [167]. The authors proposed a model PTP1B inhibitor—suramin (IC_50_ = 4.1 μM [168])—for the therapy of Parkinson’s disease (PD). Suramin was shown to decrease the neuronal damage and to reverse the locomotor deficits by preventing neuroinflammation and by the activation of antiapoptotic pathways in PTP1B-overexpressed SH-SY5Y (neuroblastoma) cells. The effect was also verified in a zebrafish PD model [167]. Furthermore, it was determined that roflumilast exerted neuroprotective effects in Parkinson’s disease based on observations of rats with rotenone-induced PD.

**Figure 8:**
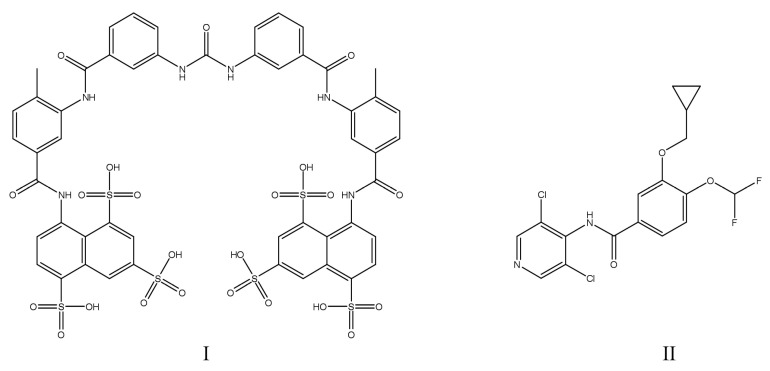
Chemical structures of PTP1B inhibitors investigated in the therapy of Parkinson’s disease: (**I**)—suramin; (**II**)—roflumilast.

This PDE4 inhibitor is typically used in the treatment of chronic obstructive pulmonary disease (COPD) and in this case acts by the indirect inhibition of PTP1B, with the effects mediated by the crosstalk between the CREB/BDNF/TrkB and SIRT1/PTP1B/IGF1 signaling pathways [169]. The chemical structures of PTP1B inhibitors that were investigated in the therapy of PD are presented in Figure 8.

Such reports are particularly interesting in relation to the abundance of this disease, as PD is the second most common type of neurodegenerative disease—less common only than Alzheimer’s disease. The symptoms of PD include impaired balance and coordination, presence of uncontrolled body movements, as well as bradykinesia, and general muscle rigidity. However, the input of PTP1B in the pathomechanism of this disease is still not completely understood and requires further investigation before drawing any definitive conclusions. More in-depth commentary regarding the potential benefits from using PTP1B inhibitors in causal treatment of neurodegenerative diseases such as AD, PD, amyotrophic lateral sclerosis (ALS), and multiple sclerosis (MS) was recently published by the authors [170].

Finally, PTP1B overexpression was also identified as a therapeutic target for the treatment of potentially life-threatening stenosis of the aortic valve induced by calcific aortic valve disease (CAVD) [171]. The exact role of PTP1B in CAVD was not established yet, but the latest reports associate this enzyme with the excessive generation of ROS and detrimental regulation of inflammatory signaling [172] in heart failure [173], as well as in the other cardiovascular diseases.

## 3. Fundamental Origins of PTP1B Overexpression

Upon discussing the involvement of PTP1B overexpression in the pathomechanisms of human diseases, it is also important to recognize the elementary origin of the excessive activity of the enzyme. Two factors were established as critical in this matter: ER stress and inflammation.

Under normal circumstances, endogenous LMO4 deactivates the PTP1B by oxidizing this phosphatase into its inactive form. This process occurs in the endoplasmic reticulum, where LMO4 is retained by palmitoylation of its C-terminal-located cysteine [104]. However, palmitoylation itself is a process susceptible to oxidation. Localized excessive oxidation known as ER stress can be induced by several factors, including an unbalanced diet and exposure to detrimental environmental conditions, such as exposure to neurotoxic metals. Therefore, these two factors are crucial in promoting the overexpression of PTP1B. Moreover, the intracellular generation of ROS induced by ER stress enhances PTP1B expression by activation of the NκBF-related signaling pathway [121].

## 4. Overview of Strategies Providing Adequate Enzyme Selectivity of PTP1B Inhibitors

All of the presented advantages of PTP1B as a molecular target, and the major role of its overexpression in the pathogenesis of numerous human diseases, justify the intensified effort being put into the development of new drugs targeting this enzyme which has been observed over the past few years. This trend is also reflected in the increasing number of reviews, which have meticulously presented these efforts and discussed strategies undertaken by researchers to explore natural active ingredients, as well as to develop brand-new drugs of semi-synthetic or fully synthetic origins [38,50,51,174,175].

According to some of the most significant conclusions, the especially desirable molecular characteristics of PTP1B inhibitors are inclusion of numerous heterocyclic moieties within the compound and the creation of highly polar drug molecules, which would interact with the highly polar active catalytic sites of the phosphatase [11]. However, due to the close structural similarity between PTP1B and TCPTP, providing adequate enzyme selectivity towards one enzyme is particularly intricate. One of the most promising solutions involves design of drugs that are capable of binding with the catalytic domains of the enzyme and simultaneously with the secondary side pockets. By achieving this goal, the drug would restrain mobility of the conserve catalytic flexible loop (also known as WPD-loop) of the enzyme [6]. Additional mechanisms to regulate the activity of the enzyme could be provided by the C-terminal tail, which affects the allostery of PTP1B by interaction with the WPD-loop [155,176,177]. Yet, it is also important to bear in mind that the inclusion of additional functional groups increases the size of the eventual drug molecule and decreases its permeability through biological barriers. This, in addition to the severity of observed side effects, proved to be a terminal problem for several investigated drugs that entered clinical trials in the past. Trials involving trodusquemine, ertiprotafib, and a few others were discussed by Liu et al. [50] and others [38,51]. Also of interest is the work of Li et al. [178], who present a systematic review of structural fragments of drug candidates that provide increased selectivity towards PTP1B.

## 5. Future Perspectives for Therapeutic Use of PTP1B Inhibitors

In line with the discussion presented in this review, the most promising perspective for the introduction of PTP1B inhibitors as therapeutic drugs seems to be their use in diseases currently devoid of established schemes of causal treatment, such as neurodegenerative diseases. Moreover, PTP1B inhibitors, based on their unique traits, could prove especially useful in challenging therapies involving breast cancers. In this case, there are several available treatment options like surgery, chemotherapy, radiation therapy, targeted therapy, or hormonal therapy. Still, the mortality of breast cancers is ever increasing and only expected to grow even more in the future [179]. Although the outlined indications are not related, in both cases the risk-to-benefit ratio associated with the introduction of a new therapeutic group of PTP1B inhibitors seems to be significantly higher than in the cases of other diseases discussed in this review. Additionally, based on the discussion presented in this work, a safe and selective antidiabetic drug acting as a PTP1B inhibitor could also prevent the onset of breast cancers by averting dephosphorylation of the JAK2/STAT3 and JAK2/STAT5 signaling pathways [180]. Such a holistic approach seems to be a completely unique feature and could prove important for this novel therapeutic group.

Another future perspective for PTP1B inhibitors is simultaneous treatment and prevention of T2DM and MDD. As previously mentioned in the corresponding sections, diabetes mellitus increases chance for depression onset, while diagnosed MDD increases the risk of T2DM onset by 37% [105]. Similarly, diseases which could be treated with PTP1B inhibitors, such as diabetes mellitus and obesity, increase the chance for neurodegenerative diseases. In this case, PTP1B inhibitors could simultaneously ameliorate metabolic disorders, as well as preventing the accumulation of amyloid plaques [72] and reducing their size in early stages of the Alzheimer’s disease [73]. Once again, the holistic approach, involving unique advantages granted by the PTP1B inhibitors, like prevention and symptomatic treatment at the same time, could prove essential for attainment of the most effective pharmacotherapeutics.

## 6. Conclusions

The outlined characteristics of selected human diseases, and the involvement of PTP1B overexpression in the pathogenesis of these disorders, suggests multidimensional mechanisms responsible for the onset of each condition. Numerous elements of these intricate mechanisms overlap to exert synergic action, or to mediate signaling pathways by negative feedback. Although the association between conditions such as T2DM and obesity seem quite apparent, their connection with mental or neurodegenerative diseases is not apparent, and better understanding of these relations may create new opportunities for the treatment of the respective diseases. And although new PTP1B inhibitors may appear appealing as seemingly versatile treatments, the inconclusive role of the enzyme in progression of certain cancer types necessitates caution towards their use in routine pharmacotherapy. Such decisions should always be based upon scrupulous risk-to-benefit analysis. The current state of the art regarding the effects inflicted by inhibition of PTP1B, especially the long-term consequences of such therapy, prevents us from drawing any definitive conclusions. However, rather than being daunting, this fact should become an encouragement towards further development within this branch of medicinal chemistry. Especially, since PTP1B inhibitors are expected to be useful in a wide array of applications, including some of the most abundant diseases, as well as in areas currently devoid of any therapeutic options.

Altogether, the information presented in this review emphasizes the amount of work still awaiting completion before fulfillment of the goal of selective PTP1B inhibitors being employed in routine pharmacotherapy. In particular, this concerns subjects such as better understanding of signaling pathways associated with the physiological role of PTP1B, the consequences of its overexpression in the pathogenesis of human diseases, as well as the relationship between the structures and activity of the drugs acting as enzyme inhibitors. However, the presented discussion undeniably validates the importance of further research, and encourages us to strive towards intensified effort in this branch of drug discovery.

## Figures and Tables

**Table 1:** List of targets presented in Figure 1 (in alphabetic order).

Abbreviation	Full Name
AKT	protein kinase B
AMPK	5′-AMP-activated protein kinase
AP-1	activator protein 1
Aβ	amyloid beta
BDNF	brain-derived neurotrophic factor
CAMK	Ca^2+^/calmodulin-dependent protein kinase(s)
DAG	diacylglycerol
ER	endoplasmic reticulum
ERK	extracellular signal-regulated kinase
FOXO1	forkhead box protein O1
GLUT4	glucose transporter type 4
GSK3	glycogen synthase kinase 3
IKK-β	inhibitor of nuclear factor kappa-B kinase subunithhv Xlh Thfbeta
IP3	inositol triphosphate
IR	insulin receptor
IRF3	interferon regulatory factor 3
IRS	insulin receptor substrate
JAK2	Janus kinase 2
JNK	c-Jun N-terminal kinase(s)
LEPR	leptin receptor
LPS	lipopolysaccharide
MAPK	mitogen-activated protein kinase
MEK	mitogen-activated protein kinase kinase
mTOR	mammalian target of rapamycin kinase
MyD88	myeloid differentiation primary response 88 protein
NF-κB	nuclear factor kappa-light-chain- enhancer of activated B cells
PDK	phosphoinositide-dependent kinase
PI3K	phosphoinositide 3-kinase
PKC	protein kinase C
PLC*γ*1	phospholipase C gamma 1
PTP1B	protein tyrosine phosphatase 1B
RAF	rapidly accelerated fibrosarcoma kinase(s)
RAS	rat sarcoma virus (G protein)
ROS	reactive oxygen species
STAT3	signal transducer and activator of transcription 3
STAT5	signal transducer and activator of transcription 5
TAU	tau protein
TLR4	toll-like receptor 4
TRIF	TIR-domain-containing adapter-inducing interferon-beta
TrkB	tropomyosin receptor kinase B

**Table 2:** Sequences of synthetic oligonucleotides that entered clinical trials as PTP1B inhibitors.

Name	Sequence
IONIS (ISIS) 113715	5′-GCUCCTTCCACTGATCCUGC-3′
IONIS (ISIS) PTP1BRx	5′-AATGGTTTATTCCATGGCCA-3′

## Data Availability

No new data were created or analyzed in this study. Data sharing is not applicable to this article.
